# Protected graft copolymer-formulated fibroblast growth factors mitigate the lethality of partial body irradiation injury

**DOI:** 10.1371/journal.pone.0171703

**Published:** 2017-02-16

**Authors:** Gerardo M. Castillo, Akiko Nishimoto-Ashfield, Cynthia C. Jones, Kasim K. Kabirov, Alexander Zakharov, Alexander V. Lyubimov

**Affiliations:** 1 PharmaIN Corp., Bothell, Washington, United States of America; 2 Toxicology Research Laboratory, Department of Pharmacology, University of Illinois at Chicago, Chicago, IL, United States of America; Northwestern University Feinberg School of Medicine, UNITED STATES

## Abstract

We evaluated the mitigating effects of fibroblast growth factor 4 and 7 (FGF4 and FGF7, respectively) in comparison with long acting protected graft copolymer (PGC)-formulated FGF4 and 7 (PF4 and PF7, respectively) administered to C57BL/6J mice a day after exposure to LD50/30 (15.7 Gy) partial body irradiation (PBI) which targeted the gastrointestinal (GI) system. The PGC that we developed increased the bioavailability of FGF4 and FGF7 by 5- and 250-fold compared to without PGC, respectively, and also sustained a 24 hr presence in the blood after a single subcutaneous administration. The dose levels tested for mitigating effects on radiation injury were 3 mg/kg for the PF4 and PF7 and 1.5 mg each for their combination (PF4/7). Amifostine administered prior to PBI was used as a positive control. The PF4, PF7, or PF4/7 mitigated the radiation lethality in mice. The mitigating effect of PF4 and PF7 was similar to the positive control and PF7 was better than other mitigators tested. The plasma citrulline levels and hematology parameters were early markers of recovery and survival. GI permeability function appeared to be a late or full recovery indicator. The villus length and crypt number correlated with plasma citrulline level, indicating that it can act as a surrogate marker for these histology evaluations. The IL-18 concentrations in jejunum as early as day 4 and TPO levels in colon on day 10 following PBI showed statistically significant changes in irradiated versus non-irradiated mice which makes them potential biomarkers of radiation exposure. Other colon and jejunum cytokine levels are potentially useful but require larger numbers of samples than in the present study before their full utility can be realized.

## Introduction

Treatments for radiation injury to the gastrointestinal (GI)-tract are desperately needed to ensure survival after exposure to ionizing radiation arising from an accident, nuclear warfare, or terrorist attacks using “dirty bombs”. It may take a day or more to mobilize medical teams and necessary life-saving drugs and equipment to the scene of a radiation disaster so treatment must be effective when given at least a day after radiation exposure. The hematopoietic system and the GI tract are the most susceptible organs to radiation injury and sufficient damage to either one can be fatal [1]. While death from bone marrow collapse occurs several days to weeks after an exposure, death from intestinal complications of acute radiation syndrome (ARS) occurs much earlier. Restoring intestinal function as soon as possible may facilitate recovery of those who would not otherwise survive and increase the effectiveness of radiological emergency responders. A class of promising agents for this purpose is the fibroblast growth factor (FGF) family. FGF7 has a potent ability to stimulate growth of intestinal epithelial and crypt cells [2–9] and is used as an anti-mucositis conditioning agent to thicken the mucosal lining prior to cancer radiation therapy. Other members of the FGF family that are potentially useful for treating radiation injury are FGF2 [2, 5] and FGF4 [3, 6, 10]. A constant high blood level of FGF4, expressed from adenovirus vector, provided survival advantage associated with both marrow and intestinal recovery after radiation exposure [3, 6]. However, the use of a viral vector is impractical in an emergency situation with an administration delay of 24–48 hours and an additional expression delay of another 24–48 hours after vector administration.

We developed several Protected Graft Copolymer (PGC) excipients that reversibly bind peptides/proteins to prevent their rapid elimination from the blood after parenteral administration, allowing for once a day or once a week administration [11–13]. The PGC-drug complex is large enough to escape glomerular elimination and to passively accumulate at sites of inflammation, infection, or radiological injury, thus potentially maximizing their efficacy. PGCs can potentially provide shelf-stability to unstable growth factors, making kits containing pre-filled disposable syringes for single subcutaneous self-administration feasible. This is of value in an emergency situation where thousands of people can be treated in the field. Such formulations could be part of first aid kits or emergency responder supplies.

In the event of exposure to ionizing radiation arising from an accident, nuclear warfare, or terrorist attacks using “dirty bombs”, it will likely take a day or more to mobilize medical teams and necessary life-saving drugs and equipment to the scene of a radiation disaster. Therefore, life-saving treatment must have robust administration, preferably not a continuous infusion, and be effective when given at least 24 hours after radiation exposure. In the present study, we developed long acting formulations of FGF4 and FGF7 for subcutaneous use (PF4 and PF7, respectively) and evaluated their ability to increase survival when given 24 hours after partial body irradiation (PBI) injury; survival would likely be increased by accelerating growth and restoration of intestinal function, which are critical in stopping electrolyte loss, sepsis, inflammation, and lack of nutrition.

Several models of gastrointestinal irradiation syndrome using Partial Body Irradiation (PBI), also called Total Abdominal Irradiation (TAI) in different species (e.g., mice and NHP), are well known and described in the literature. The percent bone marrow shielding (e.g., 0, 5, 40, 75%) has a very important impact on the model manifestation and lethality [14, 15]. The extent of bone marrow depletion influences the lethality from the loss of intestinal crypt cells [16]. However, the extent of crypt cell survival (but not lethality) was similar regardless of how radiation was delivered (Total Body Irradiation, TBI, or TAI = PBI) and whether the mice were rescued with bone marrow grafting after TBI [16, 17]. In Mason et al and Terry et al. [16, 17], it was concluded that if hematopoietic functions are maintained by PBI or bone marrow grafting, animal survival is related to the absolute number of surviving crypt cells. In our current work we employed the well-established and validated model of acute gastrointestinal syndrome with 40% bone marrow shielding proposed by Booth et al. [14]. In our study, animals had the head, forelimbs and thorax shielded (placed out of the irradiation field) which was estimated to protect approximately 40% of the bone marrow [18]. We established a full lethality curve in this model and found the LD50/30 to be 15.7 Gy.

Since PBI injury and recovery is likely associated with changes in blood biomarkers and tissue cytokines, we evaluated these changes as survival improved due to treatment with the various formulations. This is the first study that showed PF7 had a more pronounced PBI injury mitigation effect compared to other formulations tested. Additionally, this is the first study that established survival-associated biomarkers that were affected by PF4 and PF7 treatments. Continued testing of the efficacy and safety of these formulations at various doses against various levels of radiation exposure/injury will lead to a better understanding and treatment of radiation injury.

## Materials and methods

All animal studies were ethical and approved by Institutional Animal Care and Use Committees (IACUC) of the Pacific Northwest Diabetes Research Institute (Animal Welfare Assurance number: A3357-01) and the University of Illinois at Chicago (Animal Welfare Assurance number: A3460-01). All methods were carried out in accordance with relevant ethical guidelines by corresponding IACUC of each institution above.

### Mice

Male C57BL/6J and female CD1 mice (Jackson Laboratory, Bar Harbor, ME) were housed in animal facilities at the University of Illinois at Chicago (UIC, Chicago, IL) and Pacific Northwest Diabetes Research Institute (Seattle, WA), respectively. Irradiated food pellets (Diet 7912; Harlan Teklad, Madison WI or PicoLab Rodent Diet 20 5053, St. Louis MO) and water were provided to animals ad libitum from arrival until termination unless noted.

### Confirmation of the Bioactivity of FGF4 and FGF7

We used a BrdU Cell Proliferation assay kit (Cell Signaling Technology, Danvers, MA) to confirm the bioactivity of both FGF4 and FGF7 in vitro. Fibroblast 3T3 cells (ATCC CCL-163; seeded at 5x10^4 /well in a 96-well plate) in Dulbecco’s Modified Eagle’s Medium (DMEM) with 10% bovine calf serum were incubated at 37°C overnight, followed by another overnight incubation in the same medium without serum. The medium was replaced with serially diluted FGFs in the same serum-free medium and incubated at 37°C for another 24 hrs. Rhesus monkey lung epithelial 4MBr-5 cells (ATCC® CCL-208™; seeded at 2x10^4cells /well in a 96-well plate) in complete F-12K medium were incubated at 37°C overnight, followed by another overnight incubation in the same medium without serum. The medium was replaced with serially diluted FGFs in the same serum-free medium and incubated at 37°C for another 24 hrs. After 24 hrs the medium was replaced with corresponding serum-free medium containing 10 μM BrdU (150 μl/well) and then the BrDU Kit manufacturer instructions were followed.

### PGC synthesis and formulations

The PGC used for FGF7 was a copolymer with a PolyLysine backbone grafted with multiple MPEG and fatty acid as previously described by Castillo et al. [11] while the PGC for FGF4 was a copolymer with a PolyLysine backbone grafted with multiple MPEG and DTPA as described by Bogdanov et al. [19]. Binding of FGFs to various PGCs was evaluated as previously described [11]. To formulate FGF7 and FGF4, PGCs were simply mixed with the corresponding FGF at a 20:1 weight ratio in water then lyophilized.

### Pharmacokinetic profiles of FGF formulations

Lyophilized FGF4 and FGF7 with and without PGC were dissolved in saline and administered to female CD1 mice (0.6 mg FGF/kg, SC, n = 3). Plasma samples were collected with ethylenediaminetetraacetic acid at 0, 1, 2, 4, 6, 16, 24 and 48 hrs and analyzed using a FGF4 ELISA (RayBio, Norcross, CA) or FGF7 ELISA (R&D Systems, Minneapolis, MN). Pharmacokinetic profiles were analyzed using Phoenix WinNonlin software (Princeton, NJ).

### Radiation survival experiment

**Fig 1:**
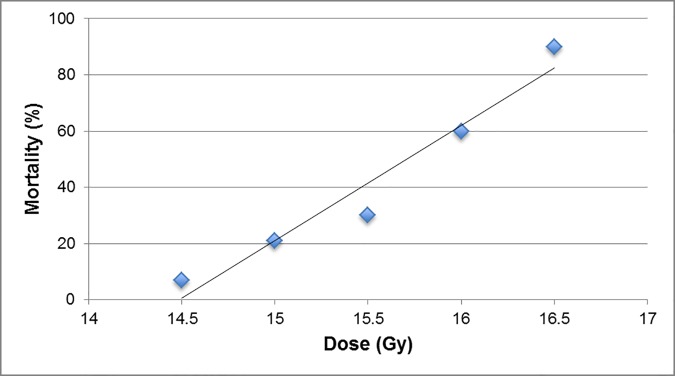
Regression line shows LD50/30 is 15.7 Gy. Shown is the % Mortality as a function of the partial body irradiation (PBI) dose received. Male C57BL/6J mice (9–10 weeks old; 14 animals per group) were irradiated at a dose range of 14.5–16.5 Gy using a 6MV LINAC photon source (Varian model #EX-21) while the head, forelimbs, and thorax were shielded. Following irradiation the animals were observed for 30 days. Mortality was seen in the range of 7–90% on days 6–8 after PBI. Probit analysis was done and percent mortality was plotted against radiation dose received (R^2^ is 0.947 indicating a very good fit). The Fisher’s exact test for data points indicate there are statistically significant differences (p<0.05) between 14.5 vs. 16.0 Gy, 14.5 vs. 16.5 Gy, 15.0 vs. 16.5 Gy, and 15.5 vs. 16.5 Gy. From these data the LD30/30, LD50/30, and LD70/30 were established at the levels of 15.3 Gy at a 95% CI of 14.912–15.687 Gy, 15.7 Gy at a 95% CI of 15.307–16.082 Gy, and 16.1 Gy at a 95% CI of 15.702–16.477 Gy, respectively. With R-squared, Fisher’s exact test, and 95% CI of LD30/30, LD50/30, and LD70/30, a high correlation is demonstrated between dose and mortality.

To establish a partial body irradiation (PBI) model, 5 groups of 9–10 week old male C57BL/6J mice (n = 14/group) were used. The 6 MV LINAC photon source (Varian model #EX-21) was used to irradiate the lower region of the body while the head, forelimbs, and thorax were shielded. Animals received the dose range of 14.5–16.5 Gy followed by 30 days of observation. Mortality was seen in the range of 7–90% on days 6–8 after PBI. Using Probit analysis we established the LD30/30, LD50/30, and LD70/30 to be 15.3 Gy at a 95% Confidence Interval (CI) of 14.912–15.687 Gy, 15.7 Gy at a 95% CI of 15.307–16.082 Gy, and 16.1 Gy at a 95% CI of 15.702–16.477 Gy, respectively (Fig 1). The 15.7 Gy PBI dose level causes LD50 in 30 days (“LD50/30”) with no deaths occurring between days 15 and 30, consistent with the data of Mason et al [16] using a PBI model. This model was also used in a subsequent study of omeprazole as a potential medical countermeasure [20]. For this radiation survival mitigation experiment, we used the same LD50/30 dose to provide maximum sensitivity to mitigation effects of various test articles.

Briefly, male C57BL/6J (9–10 weeks old) were randomized into eight groups (n = 30/group) and irradiated with 15.7 Gy. We concentrated our survival/mortality assessments up to day 15 because we expected no deaths between days 15 and 30. Animals were irradiated at a dose rate of up to 100 cGy/minute. The positive control group was treated with 300 mg/kg amifostine (UIC Hospital pharmacy) dissolved in saline and administered IP at 30 min prior to irradiation (day 0) while the negative control group remained untreated. At the time of the study design, there was no well-established and commonly accepted radiomitigator proved to be effective to mitigate GI radiation syndrome. Because our goal was to establish the GI effects of the proposed new drug formulations, we selected the well-known GI radioprotector, amifostine that is commonly used as a positive control, or comparator, in studies using GI irradiation models [21, 22]. At 24 hr after irradiation (day 1) and daily thereafter for 7 days, FGF4 (3 mg/kg), FGF7 (3 mg/kg), PF4 (3 mg/kg), PF7 (3 mg/kg), PF4/7 which contains PF4 (1.5 mg/kg) and PF7 (1.5 mg/kg), or PGC (60 mg/kg), in saline were administered SC (10 ml/kg).

Ten animals at a time were anesthetized by an IP injection of ketamine (100 mg/kg)/xylazine (5 mg/kg) to allow for immobilization. The sterna were shaved and marked to facilitate side-by-side ventral up alignment, on a Solid Water® phantom board, and accurate shielding of head, forelimbs and thorax. Animals of each set were aligned so that the lower edge of the sternum was on the upper border of the irradiation field (using a line on the Solid Water board). A second piece of Solid Water board was then placed on top, supported above and below by Lucite frames such that a minimal air space remained between the top board and the animals. Shielding was achieved by focusing the beam on this specific area of the animal from the sternum down.

The TrueBeam STx (Palo Alto, CA) LINAC was used to irradiate animals to 50% of the dose from the anterior-posterior direction (sternum to tail) and complete irradiation from the posterior-anterior direction. This provides 40% bone marrow shielding as described in the Booth et al. PBI model [18]. Dose measurements were performed with a PTW 31010 0.1 cc Semiflex Ion chamber (Freiburg, DEU) and the final dose was confirmed via nanoDot^TM^ dosimeters (Landauer Inc., Glenwood, IL) using 6 nanoDots per irradiation set of 10 animals (3 for field irradiation and 3 for out of field measurements). The nanoDot dosimeters were analyzed using the Landauer MicroStar system. Following irradiation, animals were single housed and observed twice daily for mortality/moribundity. Clinical signs and body weights were recorded once daily and 3 times a week, respectively.

The mice were monitored at least three times a day during the 15-day study period. Particular attention was given to unusually low or high activity, lethargy, shivering, appearance of fur, bloody and watery stool, and other symptoms of moribundity. The following steps were taken to minimize the suffering of the mice. Anesthesia was administered prior to irradiation and blood sample collection. Additionally, the mice were treated humanely to minimize their suffering. Mice exhibiting symptoms of moribundity were euthanized. A scoring system, assessing Body Posture (0: normal, 1: mildly hunched, 2: moderately hunched, and 3: very hunched), Activity (0: normal, 1: slightly reduced activity, 2: moving slowly, and 3: moving reluctantly/not at all) and Eye Appearance (0: normal, 1: eyes 25–50% closed, 2: eyes 50–75% closed, 3: eyes 75–100% closed) was used as the main criteria for euthanasia. Secondary endpoint criteria for moribund euthanization consisted of convulsions, ruffled coat, and a body weight loss >20%. If an animal reached a maximal score of “8” or exhibited all three of the secondary endpoint criteria, then the animal was euthanized by carbon dioxide asphyxiation followed by cervical dislocation. All surviving animals were euthanized by this method on study day 16.

### Statistical analysis

Statistical evaluation of the data produced during this study was performed to evaluate the difference between controls and drug-treated groups following PBI at expected lethality levels of LD50/30. The time to death was summarized by Kaplan-Meier plots and Kaplan-Meier estimates of the quartiles and median, when those quartiles were possible. Inter-group differences of the survival curves estimates were tested using the Log-Rank test. The proportion of subjects alive between each dose group was evaluated using the Chi-Square test. Body weights and biomarker levels were evaluated using analysis of variance tests. If a significant F ratio was obtained (p<0.05), Dunnett's t-test was used for pair-wise comparisons to the control group.

### Biomarker changes after mortality mitigation treatment

The manner in which the animals were exposed to radiation during the biomarker study was similar to the survival study above. The treatment regimen was also similar to the survival study. However for this study, only those test articles formulated in PGC were evaluated (PF4: n = 32; PF7: n = 32; and combined PF4/7: n = 36) along with controls (non-irradiated control: n = 12; PGC: n = 36; Amifostine: n = 32; and irradiated control: n = 36). The differences in n were to ensure adequate samples were available based on the expected survival. For plasma analysis of citrulline, L-FABP, and I-FABP and hematology evaluation, K_2_EDTA blood samples (from the orbital sinus) were collected from eight surviving animals per group at days 4 and 10. A GI-permeability test (n = 4/group) was done at days 4 and 10. Eight surviving animals from the irradiated groups were euthanized at days 4 and 10 to perform tissue histology and cytokine analysis. To obtain a normal control reference for tissue histology and cytokine analysis, eight animals from the normal control were euthanized at day 4. Similarly, a GI-permeability test result from this group at day 4 was used as a normal reference. Day 4 is the nadir of citrulline levels in mice [21, 23, 24] which is consistent with low crypt number based on microcolony and morphological assays [25, 26]. Day 4 was chosen because it was expected to provide the maximum possible differences, allowing us to show statistical significance from a limited number of animals. Day 6 and beyond is expected to reflect recovery, if any [21], resulting from the treatment. Day 10 was selected as a day when some markers were completely recovered and some showed signs of recovery. In our previous study of the potential use of omeprazole as a radiomitigator, complete recovery was seen in villi length by day 11 [20]. Additionally, the PBI model shows that the damage to both small and large intestines was maximal at 4 to 6 days post-irradiation, with regeneration evident on day 8, but still incomplete on day 10 [14].

### Hematology

Blood samples were analyzed for total leukocyte count (WBC) and distribution [Neutrophil (NEUT), Lymphocyte (LYMP), Monocytes (MONO), Eosinophil (EOS), Basophil (BASO)], Erythrocyte count (RBC), Hematocrit (HCT) levels, Hemoglobin (HGB) concentration, Mean corpuscular volume (MCV), Mean corpuscular hemoglobin concentration (MCHC), and Platelet count (PLT) using Hemavet 950FS Hematology system (Drew Scientific Inc., Oxford, CT).

### Citrulline assay

An Agilent 1290 Infinity LC with 6430 triple quadrupole LC-MS/MS system was used for plasma citrulline level determination. Ten (10) μL of Citrulline-d4 internal standard (IS) was mixed with 5 μL of each plasma sample in a 96-well plate. After extraction with 150 μL of 0.1% formic acid in 2-propanol and centrifugation, 100 μL of supernatant was transferred into another 96-well plate. Supernatant (5 μl) was injected onto a HILIC Silica column (Waters Atlantis®; 3 μm 2.1 × 50 mm) equilibrated with mobile phase A:B ratio of 10:90, where A is 0.1% formic acid in water and B is 0.1% formic acid in acetonitrile. Samples were eluted at a flow rate of 0.3 ml/min using a linear gradient to 50% B over 3 minutes, held at 50% B for 0.5 minutes, and re-equilibrated back in 1 min to initial composition. Citrulline and IS were detected at the retention time of approximately 2.6 min using MRM transitions 176.1 > 70.1 and 180.3 > 74.1, respectively. In addition, Arginine was monitored using MRM transition 175.1 > 70.1 (eluted at retention time 3.2 min) to ensure it does not interfere with the quantitation of plasma citrulline. A citrulline linear calibration curve (0.25 to 50 μg/mL or 1.4 to 285 μM) with 1/x weighting, origin excluded, was created in MassHunter software based on relative response (peak area of test compound/peak area of IS). All plasma samples were used without dilution as they all fit within the range of the standard curve.

### GI-permeability testing

GI permeability (n = 4/group) was tested on days 4 and 10, except for non-irradiated normal control which was only done once on day 4. For this test, an aqueous solution of Fluorescein isothiocyanate-dextran (Sigma-Aldrich, Inc., Milwaukee, WI) was administered to 16-hour fasted mice via oral gavage (13.2 mg in 0.6 ml water/mouse). After 5 hours, K_2_EDTA blood samples were collected by terminal bleed and processed to obtain plasma. Tissues from these mice were not used in further analysis. Standards (0.125–25 μg/ml), control, and plasma samples (110 μl) were placed in a black 96-well plate and fluorescence was read (485 nm excitation and 520 nm emission) using BioTek Synergy HT Plate reader (BioTek Instruments, Inc., Winooski, VT). A standard calibration curve was plotted and the sample values were then used to extrapolate concentrations from the standard curve.

### Plasma I-FABP and L-FABP ELISA assays

Mouse plasma (60 μl) was assayed for I-FABP using a sandwich ELISA Kit according to the manufacturer’s protocol (Cat # LS-F5610-1, LifeSpan Biosciences, Inc., Seattle, WA) with Peroxidase—Tetramethylbenzidine (TMB) as the reporter color generator and sulfuric acid stop solution. Mouse plasma (6 μl) was assayed for L-FABP using a sandwich ELISA Kit according to the manufacturer’s protocol (Cat # RFBP10, R&D Systems) with Peroxidase—TMB as the reporter color generator and hydrochloric acid stop solution. After stopping the reaction, the optical density (450 nm) was read using a BioTek Synergy HT Plate reader. Standard calibration curves (I-FABP 0.157–10 ng/ml; L-FABP 0.078–5 ng/mL) were plotted and the sample OD values were then used to extrapolate concentrations from the standard curve.

### Measurement of EPO, IL-18, IL-15, IP-10 and TPO from GI-Tissue

The snap-frozen mouse jejunum or colon tissues were thawed, weighed, and transferred to tubes containing Lysing Matrix D (MP Biomedicals, LLC, Santa Ana, CA). A lysis buffer containing a protease inhibitor cocktail was added to each tube at a ratio of 1:20 (volume:weight). The tubes were kept on ice and homogenized using Fast Prep-24TM 5G instrument (MP Biomedicals, LLC.) followed by centrifugation at 10,000 g for 20 minutes at 4°C. Aliquots of supernatants were analyzed on the same day, or frozen and kept at -80°C for repeat analysis, using Meso Scale Discovery (MSD) multiplex assay developed and validated at UIC. A quantitative MSD 96-well MULTI-SPOT® plate and reader Model 1250 (Meso Scale Diag., Rockville, MD) were used to simultaneously measure EPO, IL-18, IL-15, IP-10 and TPO in the same sample. The 96-well MULTI-SPOT® plate contains several electrodes/well and each electrode was pre-coated with corresponding capture antibodies. Analytes from the sample (50 μl) were captured on the surface of the electrode, then, after washing, bound analytes were sandwiched with Sulfo-Tag^TM^-linked detection antibodies or biotinylated detection antibody complexed with avidin-Sulfo-Tag^TM^. An MSD read buffer provides the reagent environment for Sulfo-Tag^TM^ facilitated electrochemiluminescence. The following capture and detection antibody pairs were used: anti-EPO (Meso Scale Diag. LLC), anti-mouse IL-18, anti-mouse TPO (R&D Systems, Inc.), anti-mouse IL-15 and IP-10 (eBioscience, Inc., San Diego, CA); along with standards: EPO (Meso Scale Diag. LLC), IL-18, IP-10, TPO (R&D Systems, Inc.), and IL-15 (eBioscience, Inc.). The amount of Sulfo-Tag complexes bound to each electrode was quantified using MSD imager that measures electrochemiluminescence emitted by each electrode. The range of quantitation of EPO, IL-15 and IP-10 was 19.5–10,000 pg/mL and the range of quantitation of IL-18 and TPO was 39.1–20,000 pg/mL. Total tissue protein concentrations were determined using a BCA kit (Pierce Biotechnology, Rockford, IL). The results were expressed in pg/ mg of protein.

### Villi length and crypt number

The entire 12 cm jejunum (~5 cm from the stomach until ~18 cm from junction to the cecum) was collected in 8 surviving animals per group on study days 4 and 10. Ten ~1cm segments from the distal end of the jejunum were processed into paraffin blocks and cut into transverse sections of full lumen circumference (5 μm thick) and stained with H&E for crypt and villus assessments for anatomy. Total height from base to tip of villi per each circumference was measured for each animal to calculate overall average villus height per dose group. The number of surviving and regenerated crypts per circumference was also assessed. An Olympus system (Upper Saucon Township, PA) was used to evaluate images.

## Results

### Binding of various PGCs to FGF4 and FGF7

To find the best pharmacokinetic (PK) extending excipient for each FGF, we evaluated binding capacities of FGF4 and FGF7 of over a dozen PGC designs. The most efficient binder for FGF4 is PGC made up of linear poly-L-lysine (PLL; 50 to 100-mer) with 35% of epsilon amino (eNH2) groups linked to methoxypolyethylene glycol (MPEG) and the remainder linked to diethylenetriaminepentaacetic acid (DTPA) [19]. This PGC with DTPA has a total capacity for FGF4 of 7:100 (FGF4: PGC) weight ratio. The most efficient binder for FGF7 is PGC with 55% of eNH2 groups of PLL linked to MPEG and the remainder linked to stearic acid [11]. This has a total capacity for FGF7 of 7:100 (FGF7: PGC) weight ratio.

### PK profiles of PF4 and PF7 are better than unformulated FGF4 and FGF7

**Fig 2:**
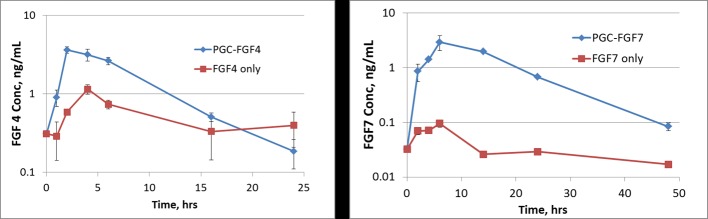
Pharmacokinetics of FGF4 and FGF7 in the presence or absence of PGC. Female CD1 mice (n = 3/ time point) were dosed subcutaneously with 0.6 mg/kg FGF4 or FGF7 without and with PGC (PF4 or PF7). Plasma FGFs were measured at various times. Based on the area under the curve, blood exposure to FGF4 (left) and FGF7 (right) is much greater in animals given PF4 or PF7 than FGF4 or FGF7 alone. PGC significantly increases relative bio-availability of both FGF4 and FGF7. Values are mean+/- SEM.

While effective diameters of FGFs are 1.5–2.5 nm, PF4 and PF7 complexes are between 22–26 nm so the PGC-FGF complexes are not subject to rapid kidney filtration/elimination (the effective glomerular pore diameter is 4 nm). After subcutaneous administration, there were significant increases in relative bio-availability and/or blood retention of these growth factors in mice (n = 3) in the presence of PGC (FGF to PGC 1:20, w/w) compared to those without PGC (Fig 2). The PK area under the curve (AUC) for FGF4 in the absence and presence of PGC was 5 h*ng/ml and 26 h*ng/ml, respectively, a five-fold increase. The AUC for FGF7 in the absence and presence of PGC was 0.2 h*ng/ml and 50 h*ng/ml, respectively, a 250-fold increase.

### FGF4 induces fibroblast proliferation but FGF7 does not

**Fig 3:**
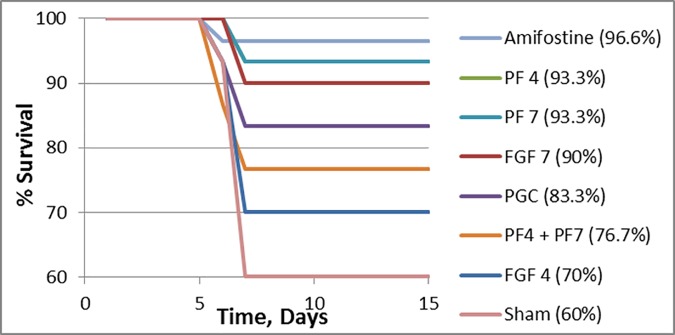
Kaplan-Meier Survival Curves of C57BL/6J Mice (n = 30/group), GI irradiated with 15.7 Gy (LD50/30). Twenty four hours after irradiation and daily for 7 days, mice were treated as indicated with 3 mg/kg unformulated FGF4, 3 mg/kg unformulated FGF7, 3 mg/kg PF4, 3 mg/kg PF7, PF4/7 which is 1.5 mg/kg PF4 plus 1.5 mg/kg PF7, or 60 mg/kg PGC carrier. Amifostine (300 mg/kg) used as a positive control was administered once 30 min prior to irradiation, and the sham irradiated control group remained untreated. Compared to the sham irradiated control (60% survival), significant survival advantage (≥90% survival) was provided by PF7, PF4, or unformulated FGF7 [p<0.02 by both Log-rank (Mantel-Cox) and Chi-square tests]. PF4, PF7, and Amifostine are not significantly different in preventing animal death. The PF4 and PF7 treated groups have higher survival than unformulated FGF4 or FGF7.

Delayed effects of acute radiation exposure include fibroblast proliferation in lung, colon, and skin. We tested the ability of recombinant hFGF4 and hFGF7 to stimulate the proliferation of non-confluent mouse fibroblast 3T3 and rhesus monkey lung epithelial 4MBr-5 cells using BrDU incorporation into DNA. The data confirmed that while FGF7 is mitogenic to 4MBr-5 with an EC50 of 1–15 ng/ml, it is non-mitogenic to mouse fibroblast 3T3 cells known to lack FGFR2b receptors. On the other hand, FGF4 is a more powerful and promiscuous growth factor that can bind to FGFR1c, 2c, 3c, or 4 receptors which are present in cells of mesenchymal tissues including fibroblast and stem cells that can differentiate into a variety of cell types [27–30]. Our data confirmed that recombinant hFGF4 has a proliferation effect on mouse fibroblast 3T3 cells with an EC50 of 1–18 ng/ml. Although both long acting formulations of these FGFs mitigated acute mortality from PBI exposure (Fig 3), a future study may reveal differences in mitigating the delayed scarring effect due to differences in receptor specificity.

### Seven-day daily administration of 5 mg/Kg PF4 and PF7 showed no clinical side effects

To balance safety and efficacy, the dose must be high enough to saturate target receptors in a sustained manner and accelerate cell/function restoration while causing no clinical signs or side effects for the duration of the treatment. The human therapeutic dose of FGF7 (Kepivance® package insert) allometrically scaled to mice is 700 μg/kg. We tested 5 mg/kg/day (7-fold higher than the human equivalent dose) for 7 consecutive days in C57BL/6J mice (n = 5) for both PF4 and PF7. During the 1-week treatment and 1-week thereafter, no clinical side effects or weight loss were observed, indicating that the dose of at least 5 mg/kg/day is well tolerated in these animals. The doses used for the radiation injury mitigation study, 3 mg/kg for individual growth factors and 1.5 mg/kg each for the combined PF4/7, were well below the tolerated dose. Future investigations may require a higher dose to see the full potential of these factors in radiation injury mitigation along with additional sub-chronic and, eventually, chronic safety evaluation of higher doses along with potential PK alteration over the course of multiple daily administration. At present, and based on our experience with PGC in general, we do not expect that PK will dramatically change using a single or multiple injections. This will need to be confirmed in future studies.

### Semiflex ion chamber and nanoDot^TM^ dosimetry indicate that animals received correct radiation dose

Each animal was targeted to have a dose of 1570 cGy from the sternum down; based on semiflex ion chamber dosimetry they received a total exposure dose of 1601.4 with an average measured exposure dose of 1594.45±3.31 cGy (mean±SD), a 1.1%– 0.1% range difference between exposure dose and measured dose. Based on nanoDot^TM^ Dosimetry all animals received infield irradiation doses ranging from 1436 to 1505 cGy (91.4% to 95.9% of intended dose) with percent average infield of 93.57±1.30% (mean ± SD). The off-target irradiation dose range was 85.97 to 127.58 cGy (5.5%– 8.1% of their target dose) with percent average out of field of 7.04±0.82% (mean ± SD) of the target dose.

### PF4 and PF7 mitigate mortality from LD50/30 PBI injury

The groups that received PF4 or PF7 showed a statistically significant 93.3% survival rate [p<0.0023 by Log-rank (Mantel-Cox) and Chi-square tests] compared to only 60% survival in the irradiated control (sham) dose group (Fig 3). Treatment with PF4/7 resulted in 76.6% survival but was not statistically significant compared to control dose group, perhaps due to dose-related lack of sustained receptor saturation. The 70% survival rate of the unformulated FGF4 group is not statistically significant while the 90% survival rate of unformulated FGF7 is statistically significant [Log-rank (Mantel-Cox) (p<0.0069) and Chi-square tests (p<0.0073)] compared to irradiated control. Most deaths occurred between days 5 and 7 and no further deaths were observed up to 15 days when all animals were euthanized. We also know that no animals will die after day 15 in the 30-day PBI model that we used (Fig 1). The efficacy of PF4 and PF7 was close to the efficacy of the Amifostine positive control with no statistically significant differences among them. The 23% improvement in survival in the PGC group relative to control is not statistically significant by Log-rank (Mantel-Cox) test (p>0.05) but is significant by Chi-square test (p<0.0449). It should be noted that PGC contains over 90% PEG by weight and PEG has been reported to inhibit free radical production [31]. Future studies with sufficient power are required to confirm the effect of PEG in this model.

### PF4 and PF7 mitigate body weight loss after PBI injury

**Fig 4:**
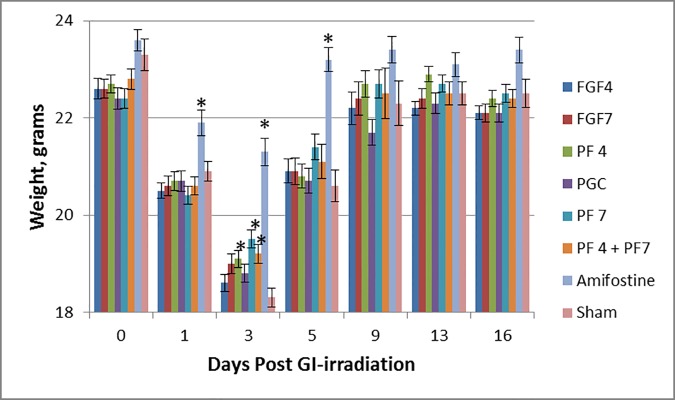
PF7, PF4, or PF4/7 mitigated radiation-induced weight loss. Although all irradiated groups lost weight, with maximum loss at day 3, groups treated with PF4, PF4 or PF4/7 showed significantly higher (p<0.05) body weight than sham irradiated control. Values are mean+/- SEM.

In general, the unmitigated irradiated sham group lost up to 22.5% of their body weight on day 3 and those that survived recovered the weight back by day 9 (Fig 4). On day 3, the mean body weights of groups that received PF4, PF7, PF4/7, and Amifostine were significantly higher (p<0.05) compared to the irradiated control group. This indicates that PF4, PF7, and PF4/7 mitigate weight loss. The conditioning agent Amifostine used prior to irradiation as a positive control was also very effective in preventing weight loss. Clinical signs such as hunched posture, sunken eyes, and decreased activity were seen in all irradiated groups throughout the study. Rough coat was seen in at least three animals in each group except the Amifostine group in which only one mouse had rough coat. The monophasic weight loss (Fig 4) is consistent with the findings of Booth et al. [14, 18]. Sufficient preservation of bone marrow (40%) in this study is unlike TBI and/or 5% bone marrow preservation which shows biphasic weight losses that are about a week apart, where the first few days of weight loss is due to gastrointestinal acute radiation syndrome (GI-ARS) followed by hematological acute radiation syndrome (H-ARS) in the second week.

### PF4 and PF7 improve hematologic parameters after GI-injury

While we expected limited damage to other organs, we evaluated hematologic changes associated with PBI injury and their potential overcompensation as a result of treatments with PF4 and/or PF7 formulations (Tables 1 and 2). This evaluation can provide a basis for future studies substantially affecting other organs including hematopoietic system. On day 4, all irradiated groups had significantly lower (p<0.05) total white blood cells (WBC), neutrophil (NEU), lymphocyte (LYMPH) when compared to the normal non-irradiated control. The significant differences in levels between the irradiated control and normal control are large enough to allow for evaluation of improvements as a result of treatment. Improvements in WBC, NEUT, LYMP, and monocyte (MONO) counts were seen in the group treated with PF7 at both days 4 and 10. At day 10, NEUT returned to baseline level or higher in groups treated with PF4 or PF7. At day 10, MONO returned to baseline level or higher in groups treated with PF4, PF7 or PF4/7.

**Table 1:** Summary of Changes in Blood and Tissue Parameters With and Without Mitigation After PBI Injury.

Test Articles	Irradiated Control vs. Normal Control		PF4		PF7		PF4 + PF7	
	Day 4	Day 10	Day 4	Day 10	Day 4	Day 10	Day 4	Day 10
**Hematology:(n = 4)**								
White Cells Count (WBC) (10^3^/mm^3^)	**87.7% ↓↓**	**74.6% ↓↓**	No	∧,*	∧,*	∧,↑,*	∧,*	∧,*
Neutrophils (NEUT) (10^3^/mm^3^)	**74.6% ↓↓**	**24% lower**	∧,*	∧,B,#,*	∧, *	∧, B, #, *	∧, *	∧, *
Lymphocytes (LYMPH) (10^3^/mm^3^)	**91.2% ↓↓**	**85.6% ↓↓**	No	∧,*	∧,*	∧,*	No	∧,*
Mononucleosis (MONO) (10^3^/mm^3^)	**61.% ↓↓**	**38.5% lower**	No	∧,B,*	∧,*	∧,B,#,*	No	∧,B,*
Eosinophil (EOS) (10^3^/mm^3^)	**60% lower**	**80% lower**	No	No	No	No	No	No
Basophils (BASO) (10^3^/mm^3^)	**50% Lower**	**0% lower**	No	No	No	No	No	No
Red blood cell (RBC) (10^3^/mm^3^)	**6.2% Higher**	**1.7% lower**	No	No	∨,$	No	No	No
Hemoglobin (HGB) (g/dL)	**5.5% Lower**	**12.4% ↓↓**	No	No	No	No	No	No
Hematocrit test (HCT) (%)	**2.75% Higher**	**3.04% lower**	No	No	No	No	No	No
Mean corpuscular volume (MCV) (fL)	**3.4% ↓↓**	**1.14% lower**	∧,↑,B,*	No	∧,↑,B,*	∨,↓	∧,*	No
Mean corpuscular HGB (MCH) (pg)	**10.8% ↓↓**	**10.9% ↓↓**	∧,↑,*	∧,*	∧,↑,*	No	∧,*	No
MCH concentration (MCHC) (g /dL)	**7.9% ↓↓**	**9.8% ↓↓**	∧,*	∧	∧,↑,*	∧,*	No	∧
Platelet (PLT) (10^3^/mm^3^)	**17.5% lower**	**52% ↓↓**	No	∧,*	∨,↓	∧,↑,B,#,*	∨,$	∧,*
**Citrulline (μM) (n = 8)**	**86.2% ↓↓**	**9.4% higher**	∧,↑	∨,B,#	∧,↑	B,#	∧,↑	∧,B,#,*
**GI Permeability (**μg/mL) **(n = 4)**	**75.4% higher**	**69.5% ↑↑**	∧,*	∨	∧,*	∨	∨	∨,↓
**L-FABP (ng/mL) (n = 8)**	**64.4% higher**	**72% higher**	∨	∨	∨,$	∧,*	∨,$	∨
**I-FABP (ng/mL) (n = 8)**	**24.5% lower**	**25.1% higher**	∨	∨	∧	∧	∧	∧
**Colon, Cytokines (n = 4) (pg/mg protein)**								
Erythropoietin (EPO)	**3.7% lower**	**52.5% higher**	∧,B,#,*	∧,B,#	∧,B,#,*	∧,B,#	∨$	∧,B,#
Interleukin 18 (IL-18)	**56.9% higher**	**89% higher**	∨,B,$	No	∨,$,	No,$	∨,$	∧B#*
Interleukin 15 (IL-15)	**25.3% higher**	**34.8% lower**	∧,B,*	∧,*	∧,B,*	No	∨,$	No
IFN-y-inducible pro. 10 (IP-10)	**93.7% ↑↑**	**89.3% higher**	No	∨,$	∨,$	No	∨,$	∨
Thrombopoieitin (TPO)	**13% higher**	**73.7% ↑↑**	No	∨,$	No	No	∨,$	No
**Jejunum, Cytokines (n = 4) (pg/mg protein)**								
EPO	**39% lower**	**48.5% lower**	∧	∧,B	∧,*	∧,B,#	∨$	∧,B,#,*
IL-18	**95.6% ↓↓**	**53.7% higher**	∧	∨,B,$	∧	∨,B,$	∧	∨,$
IL-15	**46% lower**	**41.6% lower**	No	∧,B,#	No	No	No	No
IP-10	**75.6% higher**	**19.7% higher**	∨,$	∨,$	No	∨,$	∨,$	No
TPO	**53.2% lower**	**86.6% lower**	No	No	∧,*	∧,B,#	No	∧,*
**Villus Lengths (mm) (n = 8)**	**65.7% ↓↓**	**10.7% lower**	∧,↑,*	∧,↑,B,#,*	∧,*	∧,B,*	∧,*	∧,↑,B,#,*
**Crypt Numbers (count) (n = 8)**	**95.7% ↓↓**	**53.8% ↓↓**	∧	∧	∧	∧	∧	∧

No = No Observed change compared to irradiated Control

∧ / ∨ = Observed Increase /Decrease compared to irradiated Control

B = Exceed or Return (within 1% baseline) to baseline of non-irradiated normal

# = Exceed baseline non-irradiated normal

* = Increase greater than Positive control Amifostine (>1% increase)

$ = Decrease greater than Positive control Amifostine (<1% decrease)

↑ or ↓ = Statistically significant increase/decrease compared to Irradiated Control (p<0.05)

↑↑ or ↓↓ = Statistically significant higher / lower compared to Normal Control (p<0.05).

Note: Day 7 treatment termination may account for differences between day 4 and 10 markers relative to irradiated control.

**Table 2:** Hematologic Parameters Post Gastrointestinal Radiation Injury [Mean (SD); (n = 4)].

**Group**	**Day 4**					
	WBC 10^3^/mm^3^	NEU 10^3^/mm^3^	LYMPH 10^3^/mm^3^	MONO 10^3^/mm^3^	MCVfL	PLT 10^3^/mm^3^
1 Control	8.65 (2.290)	1.50(0.442)	6.95(1.89)	0.13(0.065)	43.7(0.24)	724(65.7)
2 PF4	1.03(0.100)^1^	0.44(0.047)^1^	0.51(0.066)^1^^,^^3^	0.04(0.025)^1^	43.9(0.38)^7^	599(143.5)
3 PGC	1.50(0.556)^1^	0.48(0.179)^1^	0.92(0.332)^1^	0.06(0.024)^1^	43.4(0.68)	555(150)
4 PF7	1.48(0.419)^1^	0.50(0.132)^1^	0.86(0.261)^1^	0.08(0.021)	43.5(0.62)^7^	294(85.5)^1^^,^^3^^,^^6^^,^^7^
5 PF4/7	1.10(0.219)^1^	0.46(0.141)^1^	0.56(0.083)^1^	0.05(0.010)^1^	43.1(1.16)	516(50.7)
6 Amifostine	1.05(0.354)^1^	0.42(0.137)^1^	0.54(0.167)^1^	0.07(0.047)	43.0(0.54)	679(76.7)
7 Irradiation Control	1.06(0.285)^1^	0.38(0.119)^1^	0.61(0.152)^1^	0.05(0.010)^1^	42.2(0.36)^1^	597(226.5)
**Group**	**Day 10**					
	WBC10^3^/mm^3^	NEU10^3^/mm^3^	LYMPH10^3^/mm^3^	MONO10^3^/mm^3^	MCVfL	PLT10^3^/mm^3^
1 Control	8.65 (2.290)	1.50(0.442)	6.95(1.89)	0.13(0.065)	43.7(0.24)	724(65.7)
2 PF4	3.23(1.282)^1^	1.75(0.636)^6^	1.24(0.409)^1^	0.13(0.088)	43.0(0.39)	579(78.7)
3 PGC	2.90(0.408)^1^	1.17(0.069)	1.59(0.385)^1^	0.12(0.025)	43.8(0.74)^6^	316(46.3)^1^
4 PF7	5.75(3.554)^7^	1.83(0.259)^6^	3.67(3.217)^1^	0.21(0.134)	41.4(0.48)^1^^,^^3^^,^^6^^,^^7^	754(274.8)^3^^,^^6^^,^^7^
5 Combo	2.95(0.765)^1^	1.37(0.290)	1.43(0.891)^1^	0.12(0.043)	42.8(0.26)^3^	504(111.9)
6 Amifostine	1.75(0.646)^1^	0.84(0.343)	0.85(0.305)^1^	0.04(0.031)	42.5(0.22)^1^	455(108.6)^1^
7 Irradiation Control	2.22(0.880)^1^	1.14(0.417)	1.00(0.428)^1^	0.08(0.051)	43.2(1.12)	348(99.8)^1^

Marked significance are from Analysis of Variance followed by Dunnett’s procedure

^1^Significant Difference from Group 1 (Control) p<0.05

^3^Significant Difference from Group 3 (PGC) p<0.05

^6^Significant Difference from Group 6 (Amifostine) p<0.05

^7^Significant Difference from Group 7 (Irradiation Control) p<0.05

Note: The Day 4 data for Group 1 was used as the Control on Day 10

Mean corpuscular volume (MCV) was back to baseline or higher as early as day 4 in groups treated with PF4 or PF7 and was elevated in the group treated with PF4/7, relative to irradiated control. These improvements in MCV disappeared at day 10, 3 days after the treatments were stopped. Similarly mean corpuscular hemoglobin (MCH) improved as early as day 4 in groups treated with PF4, PF7 or PF4/7 relative to irradiated control but these improvements were not sustained at day 10 in groups treated with PF7 and PF4/7. High survival groups that received formulations containing PF7 showed decreased platelet counts (PLT) at day 4 but improved PLT at day 10 in groups treated with PF4, PF7, or PF4/7, and PLT returning to baseline level in the PF7 group. Although the number of samples analyzed (n = 4) was limited, these results indicate that these treatments have a potential to mitigate a more severe bone marrow injury which will be the subject of future study.

### Changes in potential GI-specific biomarkers and functional test after GI-injury

Blood levels of citrulline positively correlated with intestinal villi integrity while Intestinal fatty acid-binding protein (I-FABP) and liver-type fatty acid binding protein (L-FABP) have been reported to positively correlate with ongoing GI tissue necrosis [32–35]. Our results showed that citrulline entering the blood from intestinal villi is a reliable marker of intestinal integrity or number of enterocytes (Tables 1 and 2). On day 4 all irradiated groups had significantly lower (p<0.05) plasma citrulline levels than the normal control group. All groups treated with FGFs and Amifostine showed significantly higher (p<0.05) plasma citrulline levels than the irradiated control and PGC vehicle groups. At day 10 citrulline levels for all irradiated groups returned to normal except for the PGC group which significantly exceeded normal levels. This was probably a compensatory reaction of the regenerated enterocytes after being at the lowest levels (at the levels of irradiated control) compared to the drug-dosed group on day 4. Normal blood levels of I-FABP and L-FABP in C57BL/6J mice were 11±3 ng/ml and 8±0.025 ng/ml, respectively (Table 3) which are not statistically different from the irradiated groups (although a 3 to 4-fold difference was seen between the non-irradiated and irradiated groups, the variability was very high, limiting its utility at n = 8 sampling). Additionally there is a lack of consistency between survival data and changes in plasma FABP levels in this mouse study. For example on day 4, I-FABP levels in PGC and Amifostine groups are significantly lower (p<0.05) than the normal control, PF7, and PF4/7 groups, and the latter 3 groups are not statistically different from each other.

**Table 3:** Organ Related Markers and Functional Test [Mean (SD); (n = 4 or 8)].

**Group**	**Day 4**			
	Plasma Citrulline (μM) (n = 8)	GI-permeability (μg/mL) (n = 4)	Plasma L-FABP (ng/mL) (n = 8)	Plasma I-FABP(ng/mL) (n = 8)
1 Normal Control	61.7 (6.44)	0.152 (0.0502)	7.82 (0.025)	10.94 (3.008)
2 PF4	11.6 (1.98)^1^^,^^3^^,^^6^^,^^7^	1.042 (0.8145)^1^^,^^6^	12.21 (8.567)	6.48 (5.223)
3 PGC	8.5 (1.21)^1^^,^^6^	0.980(0.1697)^1^^,^^6^	8.27 (1.321)	4.87 (2.879)^1^
4 PF7	11.8 (1.85)^1^^,^^3^^,^^6^^,^^7^	0.717(0.1267)	8.17 (1.008)	11.27 (5.157)^3^^,^^6^
5 PF4/7	11.4 (2.48)^1^^,^^3^^,^^6^^,^^7^	0.581 (0.1436)	8.73 (1.883)	10.99 (3.674)^3^^,^^6^
6 Amifostine	14.5 (2.43)^1^	0.189(0.0298)	9.66 (5.236)	4.63 (1.199)^1^
7 Irradiation Control	8.5 (1.18)^1^	0.618 (0.1888)	21.99 (37.789)	8.26 (4.669)*
**Group**	**Day 10**			
	Plasma Citrulline(μM) (n = 8)	GI-permeability(μg/mL) (n = 4)	Plasma L-FABP(ng/mL) (n = 8)	Plasma I-FABP(ng/mL) (n = 8)
1 Normal Control	61.7 (6.44)	0.152 (0.0502)	7.82 (0.025)	10.94 (3.008)
2 PF4	66.7 (9.64)^3^	0.383 (0.0403)^1^	23.51 (19.383)	12.28 (5.681)
3 PGC	80.8 (10.88)^1^^,^^7^	0.428 (0.0723)^1^	12.54 (4.129)	19.15 (3.807)^1^
4 PF7	66.6 (12.87)^3^	0.398 (0.0685)^1^	50.21 (99.623)	20.79 (10.871)^1^^,^^6^
5 PF4/7	75.2 (6.24)^1^	0.378 (0.0171)^1^^,^^7^	17.48 (17.341)	16.86 (3.262)
6 Amifostine	72.9 (5.55)	0.375 (0.0451)^1^^,^^7^	8.35 (1.477)	12.27 (1.100)
7 Irradiation Control	68.1 (8.96)*	0.498 (0.0907)^1^	27.92 (55.858)	14.61 (6.948)

Marked significance are from Analysis of Variance followed by Dunnett’s procedure

^1^Significant Difference from Group 1 (Control) p<0.05

^3^Significant Difference from Group 3 (PGC) p<0.05

^6^Significant Difference from Group 6 (Amifostine) p<0.05

^7^Significant Difference from Group 7 (Irradiation Control) p<0.05

*n = 7

Note: The Day 4 data for Group 1 was used as the Control on Day 10

GI-barrier function positively correlates with Fluorescein (FITC)-dextran GI-permeability [36, 37]. After oral FITC-dextran administration, irradiated control animals had 4.2 (day 4) and 3.3-fold (day 10) higher blood FITC-dextran levels than normal control (Tables 1 and 3). At day 10, the irradiated control group had higher (p<0.05) GI-permeability than the Amifostine and PF4/7 groups while the PF4/7 group was not significantly different from all the other FGF treated groups. The results also indicate that improvement in barrier function that correlates with survival occurs much later, indicating that it is a delayed indicator of the recovery process; full recovery of barrier function in all irradiated groups was not reached at day 10 (p<0.05).

### Changes in GI-tissue cytokines

To determine if there are any robust changes detectable at n = 4 in GI-tissue cytokines that may be associated with survival as a result of treatment, we measured erythropoietin (EPO), interleukin 18 (IL-18), interleukin 15 (IL-15), interferon gamma induced protein (IP-10), and thrombopoietin (TPO) in the jejunum and colon at days 4 and 10 (Tables 1 and 4).

**Table 4:** Intestinal Cytokine levels (pg/mg protein) Post Gastrointestinal Radiation Injury [Mean (SD); (n = 4)].

**Group**	**Day 4**									
	EPO		IL-18		IL-15		IP-10		TPO	
	Colon	Jejunum	Colon	Jejunum	Colon	Jejunum	Colon	Jejunum	Colon	Jejunum
1 Normal Control	128.17(48.21)	81.43(18.65)	1467.42(1299.43)	5485.04(5307.28)	2.27(1.89)	1.61(0.95)	17.17(10.27)	21.06(14.53)	30.12(16.38)	27.74(9.105)
2 PF4	192.91(54.41)	51.90(22.70)	1465.37(1111.10)	325.18^1^(154.23)	5.66^1^(2.74)	0.72(0.10)	297.59^1^(185.17)	35.09(19.77)	46.44(17.45)	11.96^1^(5.703)
3 PGC	162.18(89.19)	21.63(31.68)	868.91^7^(633.24)	109.41^1^(100.45)	4.06(0.58)	0.53^1^(0.17)	145.63(88.67)	50.41(58.03)	39.15(15.21)	5.71^1^(6.63)
4 PF7	164.28(35.25)	61.97(59.54)	2257.31(857.67)	323.20^1^(178.14)	3.90(1.41)	0.61^1^(0.13)	130.27(51.56)	95.54(104.02)	43.68(11.18)	16.50(11.83)
5 PF4/7	90.22(26.19)	24.38(19.84)	2234.36(1330.47)	306.09^1^(235.02)	1.97(0.68)	0.77(0.33)	135.01(107.02)	70.35(22.76)	25.94(8.29)	8.10^1^(3.105)
6 Amifostine	107.91(31.60)	52.55(34.05)	2525.32(1552.31)	551.93^1^(476.03)	2.97(1.82)	0.88(0.41)	140.04(55.28)	79.60(52.12)	32.49(15.14)	14.06(5.906)
7 Irradiation Control	123.38(24.55)	49.68(42.55)	3404.48(437.51)	242.68^1^(192.47)	3.04(1.46)	0.87(0.48)	272.78^1^(222.63)	86.46(58.68)	34.62(5.46)	12.97(8.862)
**Group**	**Day 10**									
	EPO		IL-18		IL-15		IP-10		TPO	
	Colon	Jejunum	Colon	Jejunum	Colon	Jejunum	Colon	Jejunum	Colon	Jejunum
1 Normal Control	128.17(48.21)	81.43(18.65)	1467.42(1299.43)	5485.04(5307.3)	2.27(1.89)	1.61(0.95)	17.17(10.27)	21.06(14.53)	30.12(16.38)	27.74(9.105)
2 PF4	291.97^1^(108.1)	81.88(51.67)	17953.5(15542)	5897.21^6^(3830.9)	3.87^3^^,^^6^^,^^7^(2.09)	1.71(1.23)	89.95(64.40)	41.49(25.02)	103.57^1^(47.1)	20.02(10.87)
3 PGC	374.71^1^(22.3)	60.15(15.29)	16556.24(10067.7)	10252.35(4175.9)	1.56(0.22)	2.44^7^(0.79)	269.0^1^(197.51)	126.58(97.97)	147.72^1^(6.7)	21.40(13.69)
4 PF7	354.49^1^(55.52)	84.62(73.8)	13346.8(10975)	4403.28^6^(3002.7)	1.69(0.48)	0.73^3^(0.54)	162.4(58.51)	74.83(72.54)	124.44^1^(30.8)	29.72(24.80)
5 PF4/7	349.78^1^(16.23)	171.16^7^(107.01)	22172.4^1^(5464.09)	10915.26(12147.7)	1.62(0.47)	0.93^3^(0.59)	134.01(33.97)	178.27(167.11)	136.20^1^(36.7)	86.15^1^^,^^3^(55.99)
6 Amifostine	358.13^1^(136.9)	136.64(69.70)	17502.3(4134.52)	22297.51^1^(4817.8)	1.82(0.77)	1.75(0.61)	123.0(57.9)	226.55(247.07)	127.86^1^(45.7)	66.11(45.47)
7 Irradiation Control	269.90(58.90)	41.96(3.31)	13288.5(6864.9)	11836.05(9560.54)	1.48(0.33)	0.94(0.198)	161.03(94.6)	107.10(90.69)	114.67^1^(32.7)	24.03(5.37)

Marked significance are from Analysis of Variance followed by Dunnett’s procedure

^1^Significant Difference from Group 1 (Control) p<0.05

^3^Significant Difference from Group 3 (PGC) p<0.05

^6^Significant Difference from Group 6 (Amifostine) p<0.05

^7^Significant Difference from Group 7 (Irradiation Control) p<0.05

Note: The Day 4 data for Group 1 was used as the Control on Day 10

In addition to its main role in erythropoiesis, EPO is also involved in different intracellular signaling cascades in a variety of non-hematopoietic organs and tissues [38]. EPO is also implicated in anti-inflammatory and anti-oxidative effects that limit ischemia-reperfusion (I/R) injury in several tissues, including intestine [39]. These protective effects mediated by EPO have been associated with a reduction of oxygen radical concentration. The endogenous synthesis of EPO, as well as the expression of EPO receptor in non-erythroid cells, have been associated with improved tissue and organ function [38]. Currently, there is a significant body of evidence that EPO stimulates different cell types in a synchronized and coordinated manner for better survival and faster regeneration. Thus it was of interest to test EPO content in severely injured intestinal tissue at the time of potentially maximum damage and in a recovery period.

TPO was included for this analysis because TPO shares 25% homology with EPO. Besides its hematopoietic role, TPO is also known to produce biological effects in a variety of cells, including protection of cardiomyocyte from ischemia/reperfusion [40], and is also involved in endotoximeia/sepsis [41–43]. Sepsis is one of the common causes of lethality from GI radiation damage following bacteremia due to translocation of the gut microorganisms. Cuccurullo et al. [44] showed that TPO concentrations significantly increased after the induction of systemic inflammation or sepsis in two sepsis experimental mouse models (LPS model and cecal ligation and puncture (CLP) model, the latter more relevant for our study as it resembled bacterial translocation following irradiation). Moreover, these authors reported that inhibiting TPO in vivo reduced histological damage in target organs (lung, liver, and gut) in both models studied. On the other hand, increased circulating TPO may be a response to altered bone marrow hematopoiesis or bone marrow failure [45–47]. The source of TPO and EPO in intestines remains unknown. To our knowledge, this presentation is the first work which demonstrated detection of TPO and EPO in intestines. One hypothesis is that TPO is produced in endothelial cells as it is known that TPO and its receptor, c-mpl, are constitutively expressed by liver endothelial cells [48] and brain-derived endothelial cells [49]. It also may be possible that it is produced in the intestinal cells. The expression of thrombopoietin (TPO) mRNA is observed in several tissues, including liver, kidney, brain, skeletal muscle, intestine, spleen, and bone marrow [50]. It can also be from the intestinal vasculature or bleeding in the gut. An identification of the exact source of EPO and TPO will need additional studies.

On day 4, jejunum IL-18 concentrations (but not other measured cytokines) were statistically significantly decreased in all dose groups, including a 23-fold decrease in the irradiated vs. non-irradiated dose group. There was no statistically significant difference between irradiated control and normal control in the level of any measured colon cytokines, except IP-10 which was statistically significantly increased approximately 16-fold. On day 10, there was no statistically significant difference between normal control and irradiated control in the level of colon EPO, IL-18, IL-15 and IP-10 (while TPO concentrations were significantly overexpressed) and jejunum EPO, IL-18, IL-15, IP-10 and TPO content (demonstrating either complete return to normal or a tendency to return to normal). While several markers were increased and decreased several fold, the absence of statistical significance indicates high variability of the markers when sampled at n = 4. On day 4 after PBI IP-10 increased 16- and 4-fold in the colon and jejunum, respectively. IL-18, IL-15 and TPO levels were decreased in jejunum, while changed in the opposite direction in colon. Regarding the treatment, however, colon EPO dropped initially at day 4 then became overcompensated at day 10 in groups treated with PF4 and PF7 (Tables 1 and 4). At day 10, jejunum EPO concentrations were at baseline levels or overcompensated in all groups treated with FGFs. TPO levels initially decreased on day 4 in jejunum then became statistically significantly overcompensated on day 10 in PF4/7 dose group.

### Changes in intestinal villi length and crypt number after GI-injury

**Fig 5:**
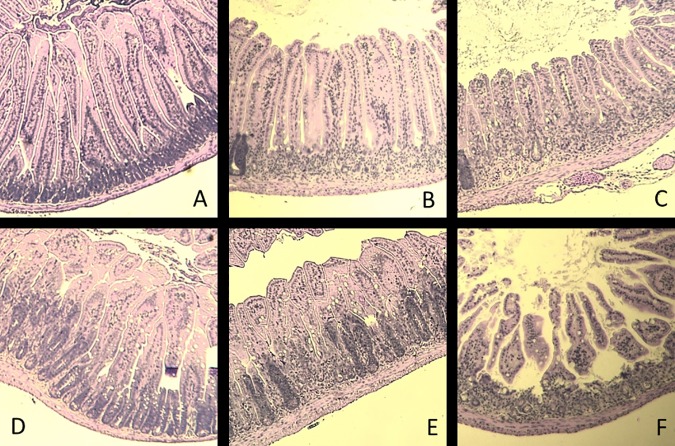
Representative Images (H&E) of Jejunum Sections at 15.7 Gy PBI in Male Mice at Day 4. A: Non-irradiated control; B: PF4; C: PF7; D: PF4 + PF7; E: Amifostine; F: Irradiated control. Pictures are 600 μm x 600 μm.

**Fig 6:**
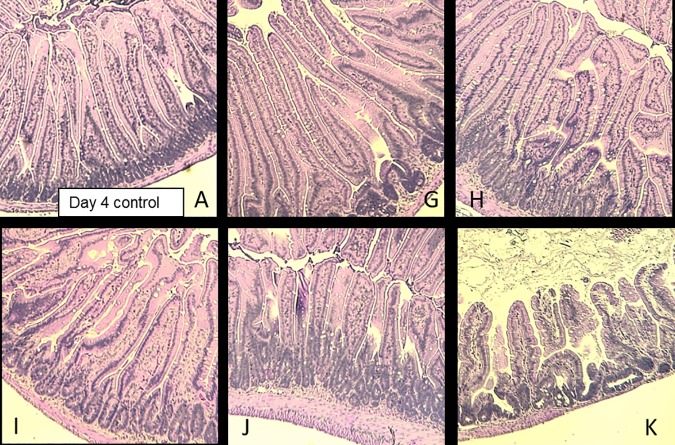
Representative Images (H&E) of Jejunum Sections at 15.7 Gy PBI in Male Mice at Day 10. A: Non-irradiated control from day 4; G: PF4; H: PF7; I: PF4 + PF7; J: Amifostine; K: Irradiated control. Pictures are 600 μm x 600 μm.

On days 4 and 10, all groups treated with FGFs had longer intestinal villi than the irradiated control group and on day 10 villus length reached or exceeded the length of the normal control baseline and Amifostine positive treatment control (Tables 1 and 5). The group treated with PF4 had significantly longer (p<0.05) villus lengths compared to the irradiated control and Amifostine positive control. On days 4 and 10, all groups treated with FGFs had greater crypt number/circumference (Tables 1 and 5, Figs 5 and 6) than the irradiated control. These changes correlate with the level of plasma citrulline and survival.

**Table 5:** Villus length and Crypt number Post Gastrointestinal Radiation Injury [Mean (SD); (n = 8)].

Group	Day 4		Day 10	
	Villus length (mm)	Crypt Number /circumference	Villus length (mm)	Crypt Number /circumference
1Normal Control	411.54 (32.57)	117 (9.7)	411.54(32.57)	117 (9.7)
2 PF4	339.42 (70.26)^1^^,^^7^	10 (4.1)^1^^,^^6^	441.39 (47.16)^6^^,^^7^	66 (5.6)^1^
3 PGC	325.96 (40.58)^1^	7 (2.8)^1^^,^^6^	411.74 (37.12)	64 (5.9)^1^^,^^6^
4 PF7	300.73 (45.81)^1^	7 (3.1)^1^^,^^6^	398.02 (56.87)	75 (23)^1^
5 PF4/7	319.20 (22.72)^1^	10 (13.3)^1^^,^^6^	425.31 (27.44)^7^	70 (5.8)^1^
6 Amifostine	295.98 (29.16)^1^	41 (9.4)^1^^,^^7^	382.30 (29.63)	82 (13.6)^1^^,^^7^
7 Irradiation Control	270.20 (61.23)^1^	5 (4.0)^1^	367.98 (42.50)	63 (6.5)^1^

^1^Significant Difference from Group 1 (Control) p<0.05

^3^Significant Difference from Group 3 (PGC) p<0.05

^6^Significant Difference from Group 6 (Amifostine) p<0.05

^7^Significant Difference from Group 7 (Irradiation Control) p<0.05

Note: The Day 4 data for Group 1 was used as the Control on Day 10

## Discussion

All 22 members [7, 51] of the FGF family are very unstable [52] and have a short biological half-life, consistent with their autocrine and paracrine role in tightly regulated organ development, which limits their practical systemic use. There are four main FGF receptors (FGFR1-4) and each can have domain IIIb (b receptors) or domain IIIc (c receptors). While FGF1 and 2 act on both b and c receptors, FGF4 acts through c receptors and FGF7 acts through b receptors, allowing us to compare the mitigating effect of stimulating these two distinct receptors. The b isoform is preferentially expressed in epithelial tissue, whereas the c isoform is expressed in all mesenchymal tissue, including fibroblast, and is also important in maintenance and differentiation of progenitor stem cell populations [28, 30, 53–56]. Despite its short half-life ([52], Kepivance® package insert), studies have shown that FGF7 is a very effective conditioning agent prior to cancer radiotherapy to prevent mucositis [8, 9, 52, 57–64]. In the present study, we used PGC to develop long acting once a day subcutaneous formulations of FGF4 (PF4) and FGF7 (PF7) to maximize the regeneration of radiation-damaged GI systems. We also showed that these long acting PF4 and PF7 formulations can mitigate lethality even if given a day after PBI. Such mitigating effects of PF4 and PF7 can be attributed to 1) its sustained systemic presence facilitated by PGC and 2) its potent ability to stimulate growth of intestinal epithelial and crypt cells [2–9]. Although PF4 and PF7 are intended to be used in life saving situations during a radiological emergency, it would still be prudent to evaluate whether a higher sub-chronic dose will have long term consequences in animals. So far no patients conditioned with short acting FGF7 have acquired new or different types of cancer afterwards. Because Adenovirus-FGF4 can maintain blood FGF4 levels above 0.16 ng/ml, use prior to PBI provides survival advantage that was clearly associated with both marrow and intestinal recovery [3, 6]. Our dosing based on the PK results (Fig 2) achieved that blood level immediately after administration and is supported by our survival data (Fig 3).

The similarity of effectiveness of either PF4 or PF7 in mitigating the lethality from PBI injury may be due to secondary local paracrine action. Systemically administered PF4 can act on c receptors of all mesenchymal lineage cells in the entire body (lymphatic, circulatory systems, connective tissues, bone and cartilage) and mesenchymal stem cells [28, 30]. Once stimulated, mesenchymal cells can produce various local acting FGFs, some of which act in an autocrine manner via c receptors and others in a paracrine manner via b receptors present in adjacent epithelial cells. The lack of specificity of PF4 may be beneficial as it may target other organs affected by radiation including bone marrow. However, such benefit needs to be balanced and tested against the long term potential for lung fibrosis, a delayed effect of acute radiation exposure, in addition to longer term safety concerns due to potential cancer. In contrast with PF4, PF7 used in the present study is shown not to be a mitogen for 3T3 fibroblast cells. However PF7 can also produce paracrine action on adjacent mesenchymal cells but such action is likely limited to the vicinity of epithelial cells. Future study is needed to evaluate the mitigating effect of these formulations after total body irradiation and to determine the organ-specific benefits provided by each formulation that contribute to survival. The PF4/7 showed survival benefits although to a lower extent compared to individual long acting formulations at higher doses (76.7% vs 93.3%), perhaps because of lower dose. Future study is needed to determine whether there is a requirement for c receptor acting FGFs to maximize survival, especially in case of total body irradiation.

With increasing bone marrow sparing, the bone marrow influence on lethality due to GI-ARS decreases and is very limited at 40%. In fact only 5% bone marrow sparing (or reconstitution of 1.8% of the bone marrow after TBI [17, 65]) is sufficient to reduce mortality during the early stage of GI-ARS in both mice and primates [14]. The entire mouse has approximately 280 million bone marrow cells [65] and 5 million represents 1.8% of the bone marrow, albeit administered systemically (thus can easily seed various sites) compared to the less mobile remaining marrow in 40% bone marrow sparing. Booth et al [14] also showed that antibiotic-preventable swollen head/muzzle syndrome due to septicemia/inflammation was normally observed in TBI and 5% bone marrow sparing, presumably from intestinal gut bacteria. However it was not seen in the 40% shielding model. Consistent with the study by Booth et al [14], we did not observe swollen head/muzzle syndrome at radiation doses that compromise the intestinal barrier while sparing 40% of the bone marrow, indicating that the remaining bone marrow may have provided protection from swollen head/muzzle syndrome. Essentially, bone marrow is critical in bacteremia and inflammation mitigation [16, 17, 66]. The acute animal lethality depends on a) the number of surviving intestinal crypt stem cells that rebuild villi enterocytes responsible for electrolyte balance and nutrition, and b) the extent of remaining bone marrow that modulates the consequences of increased intestinal inflammation and permeability that facilitate bacterial entry into the blood [16]. The 40% sparing of the bone marrow limits the bone marrow damage so that lethality is actually a result of GI-damage [17].

Jones et al. reported that higher plasma citrulline levels were significantly associated with higher crypt survival [24]. The correlation of the plasma citrulline to crypt survival was more robust for higher irradiation doses (9–15 Gy TBI) and for later time points (2–5 days post TBI). In the present study, we also evaluated the level of various potential blood biomarkers that may be associated with improvement in survival from PBI injury. The blood level of citrulline is one of the earliest and more sensitive indicators of GI-recovery and eventual survival, consistent with the measure of villus length and crypt numbers. However, despite the survival-predictive increase in citrulline levels, correlated to villus length and some non-statistical increase in crypt numbers at day 4, the GI-permeability did not show statistical improvement until day 10. Some GI-permeability improvement in the PF4/7 dose group, compared to irradiated control and especially PGC vehicle, was evident as early as day 4 but these groups show only modest survival advantage (76.7% and 83% respectively) compared to 93% for PF4 and PF7. This indicates that the enterocyte population increases significantly prior to the full restoration of the crypt and intestinal barrier function, which were not completely restored at day 10. Barrier function is critical for electrolyte balance and prevention of inflammation and sepsis from gut endotoxin and bacteria.

In this PBI study in mice, the levels of I-FABP and L-FABP were quite variable and were not very sensitive markers of GI-injury and recovery. Although plasma I-FABP is associated with intestinal necrosis [35], its value as a radiation injury biomarker is limited [32], perhaps because the release of I-FABP occurs acutely and may fluctuate over time, along with a limited half-life of about 20 min [67, 68]. Area under the curve over time of FABPs in blood may provide much better feedback. Another cause of variability and lack of sensitivity could be the ELISA kits used; previous studies in monkeys used MSD assays developed and validated at UIC which showed the high utility of these tests (data not published).

In the present study, improvement in WBC, NEUT, LYMPH, MONO, and PLT counts as well as the MCV, MCH, and MCHC appears to be associated with improved survival. They may be even more important markers of recovery for total body irradiation with more significant bone marrow involvement and we recommend measuring them for subsequent evaluation of PF4 and PF7 effects in a hematopoietic acute radiation model.

The pattern of PBI-induced cytokine changes is different for colon and jejunum (Table 1) and the FGF treatments appear to be suppressing inflammation, as indicated by the decrease in IL-18 and IP-10. We observed a limited initial decrease in GI-tissue EPO while TPO is variable in the irradiated control. Treatment with PF4, PF7, and PF4/7 resulted in an increase above normal levels in most cases (Table 1), indicating potential utility in total body radiation injury. IL-18 concentrations in jejunum as early as day 4 and TPO levels in colon on day 10 following PBI showed statistically significant changes in irradiated vs. non-irradiated mice which makes them potential biomarkers of radiation injury and recovery. Significant changes in IL-18 following irradiation insult to the GI correlates well with its well-known critical roles in intestinal host-microorganism homeostasis and inflammation [69]. Other colon and jejunum tissue cytokine levels require larger number of samples than the present study before their full utility can be realized.

In addition, an increase in TPO levels correlates to an increase in PLT in the PF-dosed group. It is not clear if TPO and EPO are from circulating blood present in the blood vessels of the intestinal tissues or may be synthetized in it (although no reports about this latter possibility were found by us in the literature). This will be the subject of a future study. The results of this study indicate that blood citrulline levels, crypt numbers, and GI-permeability are reasonable parameters for assessing GI-recovery associated with survival. All these biomarkers showed improvement following treatment with our drug formulations, although to different extents at the selected time-points. These biomarker improvements likely contributed to increased survival through improvement in nutrient absorption and barrier function, which prevents bacteremia/septicemia. In addition to blood citrulline levels, both increased crypt number and permeability improvement were associated with survival in this study. The crypt numbers were higher (although not statistically significant in any group other than the positive control group) in all drug-dosed groups compared to the irradiated control group without drug on day 4. There was as high as a 2-fold increase in crypt numbers in the PF4/7 group compared to the irradiated control. Consistent with the study described by Booth et al. [14], we did not see a complete recovery of crypt numbers by day 10 which corresponds to incomplete recovery of permeability function. While not completely recovered by day 10, permeability improved with drug treatment and was most evident in the significant decrease in permeability in the PF4/7 treated group compared to irradiation control. The PF4/7 group also showed the lowest permeability on day 4 (not significantly different from normal control group). The recovery of in vivo permeability and crypt counts was a later event (not recovered completely by day 10 after irradiation) relative to the blood citrulline concentrations and villus length. Although crypt numbers increased in all drug-dose groups compared to the irradiated control group, the lack of statistical differences may be due to the high variability of the crypt number between animals, especially in the early time-points when only a few crypts survived. Perhaps differences in recovery in crypt numbers between the drug-dosed and untreated irradiated groups would be evident during the critical days of animal death (days 7–8) which were outside the goals of this study. Establishing mechanistic histological events will take more animals and multiple time-points along with a comparison of the crypt counts in surviving vs. dead animals. In this study, we showed that our drug formulations were effective in improving survival following a lethal dose of irradiation and we defined survival-associated biomarkers and the inherent variability in other biomarker candidates. This study will guide our future assessment of other radiomitigator candidates.
